# Thermodynamic Evaluation of a Triple-Pass Reverse Osmosis Seawater Desalination Plant: Energy and Exergy Perspectives

**DOI:** 10.3390/membranes16070227

**Published:** 2026-07-01

**Authors:** Abdulrahman S. Almutairi, Hani Abulkhair, Saad F. Almokmesh, Talal E. Alotaibi

**Affiliations:** 1 Department of Mechanical Power and Refrigeration Technology, College of Technological Studies, Public Authority for Applied Education and Training, Shuwaikh, P.O. Box 42325, Kuwait City 70654, Kuwait; sf.almokmesh@paaet.edu.kw (S.F.A.); te.alotaibi@paaet.edu.kw (T.E.A.); 2 Department of Mechanical Engineering, Faculty of Engineering, King Abdulaziz University, P.O. Box 80204, Jeddah 21589, Saudi Arabia; haboalkhair@kau.edu.sa; 3 Center of Excellence in Desalination Technology, King Abdulaziz University, P.O. Box 80200, Jeddah 21589, Saudi Arabia

**Keywords:** reverse osmosis, triple-pass desalination, exergy, recovery ratio, energy recovery device

## Abstract

Energy and exergy analyses were conducted on a triple-pass seawater reverse osmosis desalination system to evaluate thermodynamic performance and identify primary sources of irreversibility. A comprehensive simulation model, developed in IPSEpro (Version 7.0) and validated against manufacturer data, demonstrated strong agreement with the reported values. Exergetic efficiency of the reverse osmosis (RO) units increased across the passes, from 57% in the first pass to 80% and 78% in the second and third passes, respectively, while exergy destruction decreased correspondingly from approximately 375 kW in the first pass to 120 kW and 130 kW in the second and third passes. The pumping system, particularly the main high-pressure pump, was responsible for 49% of total exergy destruction, followed by the first RO unit at 23%. The impacts of feed water temperature, high-pressure pump pressure, and water recovery ratio (RC) on exergetic efficiency, specific energy consumption, and permeate flow rate were systematically assessed. Increasing the feed water temperature from 15 °C to 33 °C enhanced exergetic efficiency from 27.8% to 29.9% and reduced total exergy destruction from 1622 to 1582 kW, supporting the integration of hybrid RO-thermal desalination systems. The first-pass recovery ratio emerged as the most influential operational parameter overall, with exergetic efficiency rising from 25.1% to 33.7% as RC_1_ increased from 0.35 to 0.60. Analysis of the overall recovery ratio identified RC = 0.39 as a practical operating target that balances specific energy consumption of 4.05 kWh/m^3^ and exergy destruction of 1700 kW, offering the most favourable compromise between energy efficiency and thermodynamic performance. The results presented here provide practical guidance and recommendations for the optimization of the performance of large-scale multi-pass reverse osmosis seawater desalination plants.

## 1. Introduction

A major challenge facing the 21st century is the scarcity of potable water in the face of rapid population growth, urbanization, industrial expansion, and, increasingly, the impact of climate change [1,2]. The availability of clean, drinkable water is at the forefront of concerns regarding international sustainability, with natural freshwater resources being depleted at an alarming rate [3]. While some 70% of Earth’s surface is covered by water, only a small proportion is potable and available for immediate use [4]. Increased pressure on conventional water resources has meant widespread over extraction, resulting in a significant proportion of the global population being under conditions where local water resources may be insufficient to meet demand. According to UN-Water, approximately 4 billion people experience severe water scarcity for at least 1 month each year, while over 700 million lack reliable access to safe drinking water [5]. In areas where precipitation and groundwater resources are not sufficient to meet growing domestic, agricultural, and industrial demand, such as the arid and semi-arid regions of the Middle East and North Africa, these challenges are particularly acute [6]. The severity of the challenge has meant that desalination is no longer an option but a necessity to ensure water security and stability of the long-term supply. Desalination can play a vital role in water-scarce regions by utilizing the vast available reserves of seawater and brackish water to provide a climate-resilient solution that can meet the foreseen future demand for both domestic and industrial water, decoupling local water supply from the traditional natural hydrological cycle [7,8].

Among the currently available desalination technologies, Reverse Osmosis (RO) has become the dominant desalination technology worldwide due to its relatively low energy consumption, operational flexibility, and its ability to readily adapt to technological advances [9]. However, the RO desalination process, although an indispensable solution to global freshwater scarcity, is heavily energy-intensive, so that improving its thermodynamic performance is an important area for ongoing research [10]. Conventional analysis using the First Law of thermodynamics quantifies only total energy exchange and is incapable of locating and quantifying the precise sources of inefficiencies [11]. Exergy analysis, based on the Second Law, can overcome these limitations of First Law analysis by quantifying and mapping the magnitude and distribution of irreversible losses in each component, such as the high-pressure pumps, membrane modules, and energy recovery devices (ERDs) [12,13]. This method facilitates improvements in the desalination process via two primary effects: (i) targeting component-level optimization by isolating dominant irreversibilities and (ii) rigorously linking thermodynamic efficiency and environmental sustainability, because minimizing exergy destruction will directly reduce primary energy demand and associated carbon emissions [14,15,16].

Numerous researchers have conducted exergy analyses of RO desalination systems because exergy analysis provides a comprehensive framework for the systematic evaluation of performance and optimization of the design of advanced desalination systems. Published research has consistently identified membrane modules, high-pressure pumps, and throttling valves as the principal sites of irreversible processes [17,18,19,20]. Reported exergetic efficiencies have generally been low, ranging between 4.1% and 5.82%, reflecting a significant thermodynamic gap between actual operation and the reversible limit [21]. Incorporating energy recovery devices, particularly the Energy Recovery Turbine (ERT) and Pressure Exchanger (PX), which recover waste pressure energy from the high-pressure brine reject stream, can decrease total power consumption by up to 50% and improve exergetic efficiency by up to 77%, with the PX demonstrating superior performance in both metrics [22,23]. Advanced configurations, such as pressure-retarded osmosis (PRO) integrated as an energy recovery device, have theoretically shown the potential to increase exergetic efficiency from below 2% to approximately 20%, while reducing net input power by 38% [24]. Such findings confirm that optimization at the component level combined with advanced energy recovery is likely to represent the most effective strategy for enhancing the thermodynamic efficiency of RO desalination plants [25]. Mathematical modelling of RO systems varies from simple lumped parameter methods, which treat the membrane system as a black box, to detailed distributed models that show local differences along the membrane. The simple methods are fast to compute but do not show details of each part, while the detailed models are very accurate but more complex. In this study, a detailed middle-ground model is used, giving enough detail for component-level energy analysis while still being manageable for sensitivity testing. There have been a number of recent studies investigating exergy destruction in water purification systems using single- and double-pass reverse osmosis. All have reported low second-law efficiencies, and all have identified brine disposal, high-pressure pumps, membrane modules, and pressure control valves as the major sources of irreversibilities [26,27,28]. However, as yet, no exergy analysis has been carried out on a triple-pass reverse osmosis (TPRO) seawater desalination plant. This is a significant gap in current knowledge because while triple-pass configurations are expected to form a significant proportion of new RO plants, due to the interactions between the passes, their complexity is greater than simply adding a separate additional membrane stage. Each of the three passes will operate at its own pressure and salinity level, but will interact with the other stages due to the brine recirculation between them. In addition, there will be multiple energy recovery devices situated at relevant points. All of which will generate new patterns of irreversibility with efficiency trade-offs that cannot be predicted from single or double pass studies.

Typically, with double-pass systems, the first pass provides the major salt reduction, and the second pass treats relatively low-salinity permeate with lower exergy destruction than the first pass. However, with TPROs, the recovery ratios and pressures across each of the three passes are interdependent, which complicates the thermodynamic behavior so that each pass requires its own dedicated analysis. Here, we address the gap in present knowledge by presenting a comprehensive exergy analysis of a TPRO seawater desalination plant. We quantify exergy destruction for each component, assess overall second-law efficiency, and identify the principal sources of thermodynamic irreversibility. These findings are intended to provide data to underpin improvements in the design and operation of advanced multi-pass RO desalination systems.

## 2. Reverse Osmosis Desalination Plant

**Figure 1:**
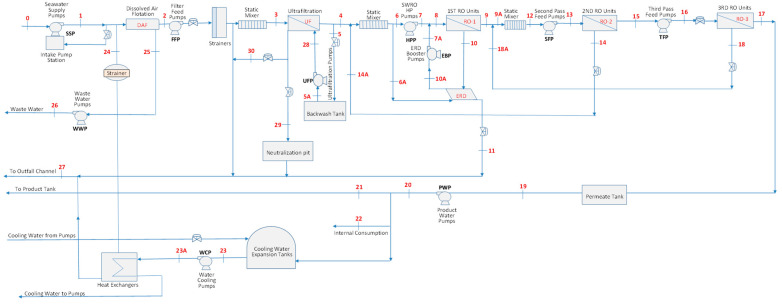
Schematic diagram of the triple-pass reverse osmosis seawater desalination plant.

The RO desalination system analysed here is inspired by and modelled on, but is not intended to exactly replicate the triple-pass seawater reverse osmosis (SWRO) Rabigh desalination plant in Saudi Arabia. This plant draws its feedwater from the Red Sea, one of the world’s most hostile feed environments with salinity levels as high as 41,000 ppm and an electrical conductivity of approximately 59,000 μS/cm. The feed water composition of the Red Sea is primarily characterized by sodium chloride (NaCl), which represents the largest proportion of dissolved salts. Additionally, significant concentrations of magnesium (Mg^2+^), calcium (Ca^2+^), sulfate (SO_4_^2−^), potassium (K^+^), and bicarbonate (HCO_3_^−^) ions are observed [29]. These dissolved ions affect osmotic pressure, scaling potential, and the overall performance of reverse osmosis (RO) membranes. Consequently, a thorough evaluation of the ionic composition of Red Sea seawater is essential for accurately assessing desalination plant operation and performance. The plant consists of 16 RO trains, of which 14 operate continuously at a nominal rate of some 500 m^3^/h each, with two on standby, which gives a capacity of 168,000 m^3^/day and a peak of 192,000 m^3^/day. A schematic representation of the overall process configuration is presented in Figure 1. A coastal intake system draws raw seawater into a dissolved air flotation (DAF) unit, where physical and chemical pretreatment removes colloidal suspensions, suspended oils, and grease. Filter feed pumps (FFPs) then pass this pretreated water through strainers and a static mixer, after which it enters an ultrafiltration (UF) system, which uses membranes with pore sizes significantly larger than those in the downstream RO membranes to remove bacteria, colloids, macromolecules, and proteins. The resulting UF output is then pressurized by the first pass high-pressure pumps (HPPs) to about 6.4 MPa before it enters the first RO stage (RO-1). The first pass is the SWRO stage, which uses cellulose triacetate hollow fiber membranes, chosen for their long-term operational stability under the conditions found in the Red Sea. Each RO-1 train processes an inlet feed of around 1327 m^3^/h with a typical S value of 59,000 μS/cm, achieving a 43% water RC_1_, with a permeate stream for each train of 570 m^3^/h, having an electrical conductivity of 280 μS/cm. The brine stream of some 757 m^3^/h is passed through an ERD, where hydraulic energy carried by the fluid is recovered and transferred from the high-pressure concentrate to the incoming feed stream, thus reducing the net specific energy consumption of the first pass. The RO-1 permeate is fed to the second pass brackish water reverse osmosis (BWRO) units (RO-2), which operate at a feed pressure of approximately 0.9 MPa and use low-pressure polyamide spiral wound membranes with high salt rejection efficiency. RO-2 feed flow per train is about 626 m^3^/h. With RC_2_ of 90%, 564 m^3^/h of permeate is output with electrical conductivity 26 μS/cm, equivalent to a Total Dissolved Solids (TDS) of about 13 mg/L. The rejected brine stream, of some 62 m^3^/h, has a conductivity of 2500 μS/cm. No second pass ERD is employed in this configuration, so no energy is recovered from the discharge. The permeate stream from the first and second passes is fed to the third pass freshwater reverse osmosis (FWRO) units (RO-3), which are the final improvement stage. The RO-3 units operate with a feed pressure of about 1.0 MPa and an RC_3_ value of 90%. Each train produces 508 m^3^/h of permeate with an electrical conductivity of 11 μS/cm, corresponding to a TDS of some 5.5 mg/L, which meets the stringent specifications for water for high purity applications: TDS < 10 mg/L and Cl^−^ < 5 mg/L.

The third pass rejected brine of volume flow rate about 56 m^3^/h with conductivity 170 μS/cm is discharged into the outfall channel. Finally, the permeate from all three passes is collected in a permeate tank and pumped to the product storage tank by the product water pumps (PWPs). Waste streams, including DAF sludge and UF backwash water, are collected and discharged via wastewater pumps (WWPs). Thermal control is maintained via a closed-loop water system driven by water cooling pumps (WCPs).

## 3. Assumptions

The proposed reverse osmosis seawater desalination plant model was developed based on the following assumptions:

1. The RO desalination plant operates under steady state conditions.
2. The effects of potential and kinetic energy are neglected throughout the system.
3. All pumps and pressure exchangers operate adiabatically with no heat loss to the surroundings. All pumps are modelled with constant isentropic efficiency values.
4. The RO membranes are assumed to be perfectly impermeable to dissolved salts, with salt passage considered negligible.
5. The pressure drop across the RO membrane modules is assumed to be constant along each pass.
6. Piping heat losses and pressure drops in connecting pipelines are considered negligible.

## 4. Second Law Analysis

The second law of thermodynamics primarily addresses the irreversibility in energy systems. Second law analysis is a useful tool for determining the irreversibility of various processes and engineering components, as well as for quantifying the maximum available work. Several studies in the literature reported that the second law analysis is presented in different approaches based on understanding the second law of thermodynamics by engineers and scientists [30]. Exergy analysis is one of the most popular approaches based on the second law of thermodynamics. Exergy is the maximum useful work that can be produced by the system before it reaches equilibrium with a specified reference environment. Exergy is conserved within a component or system when a reversible process is implemented, whereas it is destroyed in an irreversible process. The exergy balance equation is expressed as follows:

(1) $$\sum (1-\frac{T_{o}}{T_{k}}){\dot{Q}}_{k}-{\dot{E}}_{w}\;=\;\sum_{out}\dot{m}e-\sum_{in}\dot{m}e+{\dot{E}}_{d}$$

where the subscripts o and k represent the reference state and the component within the system. The left-hand side of Equation (1) represents exergy transfers by heat and work, while the right-hand side refers to the exergy transfers at inlets and outlets with exergy destruction within the component ${\dot{E}}_{d}$.

Neglecting the kinetic, potential, electrical, magnetic, nuclear, and surface tension effects leads to the total exergy consisting of two components: physical and chemical exergies [31,32,33]. The general exergy balance of a system can be written as:

(2) $${\dot{E}}_{x}={\dot{E}}_{ph}+{\dot{E}}_{ch}$$

The maximum useful work that can be derived from a unit mass of a substance when it transfers from a specified state to the reference state is called the physical exergy, and is given by the expression:

(3) $${\dot{E}}_{ph}=\dot{m}\;\left[\left(h_{s}-h_{o}\right)-T_{o}(s_{s}-s_{o})\right]$$

The specified state and reference state are represented by subscripts *s* and *o*. The maximum energy that can be produced when mass flows from a reference state to a dead state due to concentration differences is represented by chemical exergy and is given by the following expression for a gas mixture, a fuel, and a saline water stream.

(4) $${\dot{E}}_{ch,w}=\dot{m}\sum w_{k}(\mu_{k}^{s}-\mu_{k}^{o})$$

The thermodynamic and chemical properties of the working fluid play a fundamental role in determining the performance of energy systems, particularly in seawater desalination processes, where accurate property estimation directly influences the reliability of simulation and optimization results. In the open literature, four principal approaches have been proposed to evaluate the thermophysical properties of saline water: (i) ideal solution assumptions, (ii) empirical correlations derived from experimental data, (iii) equations of state, and (iv) the International Association for the Properties of Water and Steam (IAPWS) standard formulations. Among these, empirical correlation based approaches offer a favourable balance between computational efficiency and physical accuracy across the salinity and temperature ranges encountered in RO desalination practice. Accordingly, the most recent thermophysical property correlations for saline water are adopted in the present study, as reported by [34]. These correlations express the density, specific enthalpy, specific entropy, and specific Gibbs energy of seawater as explicit functions of temperature T (°C) and salinity $w_{s}$ (gs/kgsw) or (kgs/kgsw). The full set of equations and corresponding empirical constants is detailed in Appendix A.

Chemical potential is a fundamental intensive thermodynamic property that quantifies the tendency of a component to migrate from one stream to another. Constituent particles naturally flow from regions of higher to lower chemical potential, driven primarily by compositional differences, until equilibrium is reached and the chemical potential becomes uniform across all coexisting phases. In the context of seawater desalination, the chemical potentials of the water and salt components are derived from the partial derivatives of the total Gibbs free energy function with respect to the molar quantity of each constituent at constant temperature, pressure, and composition, yielding the expressions given in Equations (5) and (6), respectively:

(5) $${µ}_{w}=\frac{\partial G_{sw}}{\partial m_{w}}=g_{sw}-w_{s}\frac{\partial g_{sw}}{\partial w_{s}}$$

(6) $${µ}_{s}=\frac{\partial G_{sw}}{\partial m_{s}}=g_{sw}+{(1-w}_{s})\frac{\partial g_{sw}}{\partial w_{s}}$$

Reducing the work input required to separate salt from saline water is one of the central objectives in desalination research and system optimization. For a steady flow process operating under adiabatic conditions, the theoretical minimum separation work can be evaluated using the following expression:

(7) $${\dot{W}}_{min}={\dot{E}}_{brine}+{\dot{E}}_{product}-{\dot{E}}_{feed}$$

Exergetic efficiency evaluates desalination plant performance from a thermodynamic perspective and requires specifying the product and fuel, as exergy analysis depends on energy quality. In desalination, it is the ratio of the minimum separation work (product exergy) to the total energy input (fuel exergy) supplied, and is given by the expression:

(8) $$\eta_{ex}=\;\frac{{\dot{W}}_{min}}{{\dot{E}}_{f}}$$

For every component within the system, the rate of exergy leaving the control volume is inevitably lower than the rate entering, owing to irreversibilities associated with exergy destruction and exergy losses to the surroundings. Under steady state operating conditions, the relationship between these quantities is governed by the following expression:

(9) $${\dot{E}}_{i}={\dot{E}}_{e}+{\dot{E}}_{d}+{\dot{E}}_{L}$$

where ${\dot{E}}_{d}$ and ${\dot{E}}_{L}$ represent the rate of exergy destruction associated with internal irreversibilities within the component, and the rate of exergy loss transferred to the surroundings, respectively. Table 1 presents the exergy destruction rates and exergetic efficiency expressions for the main components of the proposed system under steady state operating conditions. The exergetic efficiency is defined for each component as the ratio of the product exergy to the fuel exergy, providing a measure of thermodynamic performance and identifying the sources of inefficiency within the system.

The performance of the triple-pass seawater reverse osmosis desalination system is evaluated using key thermodynamic and operational parameters. The recovery ratio (RC) represents the fraction of feed water (${\dot{m}}_{f})$ converted into permeate (${\dot{m}}_{p})$ and is defined as:

(10) $$RC=\;\frac{{\dot{m}}_{pr}}{{\dot{m}}_{fe}}$$

The specific energy consumption (SEC) measures the electrical energy a system must expend to produce each cubic meter of permeate, expressed in kWh/m^3^, and takes the form:

(11) $$SEC=\;\frac{{\dot{W}}_{total}}{{\dot{m}}_{pr}}$$

Taken together, these parameters expose how efficiently the system converts hydraulic input into separation work, how reliably the membrane sustains solute rejection under imposed flux, and how closely actual thermodynamic behavior tracks theoretical predictions as operating conditions shift.

## 5. Results and Discussion

**Figure 2:**
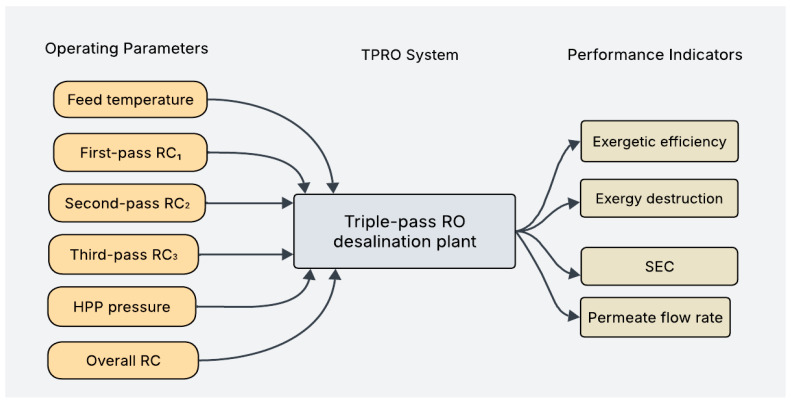
Overarching framework of the study mapping the relationship between independent operating variables and dependent performance criteria.

Here we present the energy and exergy analyses for the TPRO seawater desalination system. The dead state was defined as the winter seawater feed conditions: T_0_ = 15 °C, P_0_ = 101.325 kPa, and ws = 41,000 ppm. Figure 2 outlines the analytical framework adopted in this study, showing how the investigated operating parameters are linked to the resulting performance indicators.

Before analyzing the TPRO performance, the simulation model was validated against test data supplied by the manufacturer [35]. Table 2 compares simulated and measured results for all three passes under identical conditions. The model showed strong agreement with measurements, with an average difference of only 1.40% and a maximum difference of 3.87%, confirming it as an accurate representation of the plant’s fluid flow and mass-transfer behavior. Table 3 lists thermodynamic properties at selected state points: mass flow rate, pressure, salinity, specific exergy, and temperature, which form the basis for the exergy destruction and exergetic efficiency analyses in the following sections.

**Figure 3:**
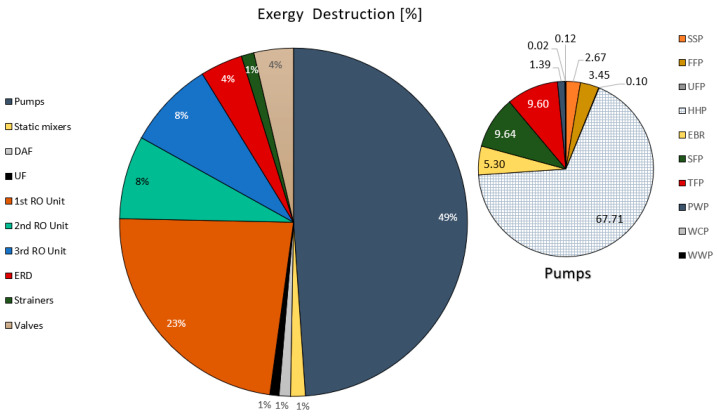
Exergy destruction as a proportion of total exergy destruction for the main components in a triple-pass reverse osmosis seawater desalination plant.

Figure 3 presents the exergy destruction in the main components of the triple pass reverse osmosis seawater desalination plant as a percentage of the whole. As shown, the largest share is due to the pumping system, about 49%, highlighting the impact of the irreversibilities in the pressure-driven membrane process. The main high-pressure pump (HPP) accounts for some two-thirds (67.71%) of total exergy destruction (Ėd) in the pumping system; next come the second and third pass feed pumps, totaling almost a fifth of Ėd (9.64% and 9.60%, respectively); the remaining 13% of Ėd is divided between the seven auxiliary pumps. This distribution is as would be expected, since the HPP will be operating with the greatest pressure difference because it has to overcome the osmotic pressure of the concentrated seawater feed, which will result in the generation of significant entropy due to hydraulic losses due to having to overcome viscous forces. The second largest contributor to the Ėd is the first RO unit, which accounts for just over a fifth (some 23%), due largely to inherent irreversibilities incurred during membrane separation. These include concentration polarization, hydraulic resistance, and salt diffusion. The second and third RO units both contribute just under 10%, due to lower operating pressures and reduced salinity in their feeds. Energy recovery (ERD) accounts for about 4% of overall exergy destruction, due primarily to fluid friction and the mixing of the high-pressure concentrate and low-pressure feed water in the pressure exchanger. A further 8% is accounted for by other components: the DAF and UF units, strainers, valves, and static mixers. This shows that pretreatment and post-treatment units have little effect on exergy destruction compared to the pumping systems and RO membrane units. These findings show that the exergy destruction is dominated by pressure-driven, not thermal losses. It follows that the first steps to reduce overall exergy destruction should be in the selection of highly efficient pumps, such as variable speed, high-pressure pumps equipped with variable frequency drives. The second would be improved membrane pretreatment and implementation of more effective cleaning strategies to reduce both fouling and concentration polarization. The third step would be to upgrade to an ERD with lower mixing and friction losses, such as an advanced isobaric pressure exchanger. Certainly, all three steps would be taken simultaneously.

**Figure 4:**
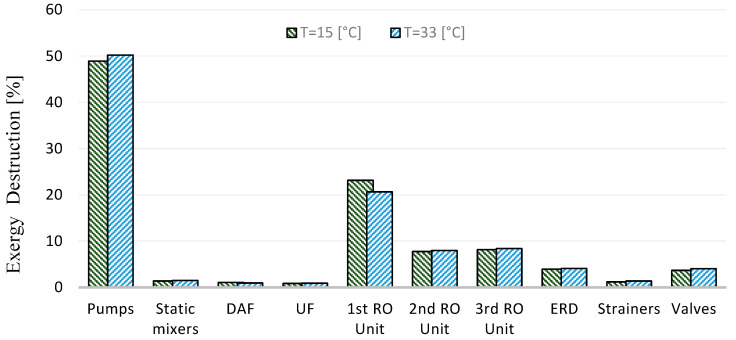
Exergy destruction as a proportion of total exergy destruction across system components under summer and winter seawater inlet temperature conditions.

Figure 4 shows the effect of feed water temperature on the exergy destruction in the main components of the triple pass reverse osmosis seawater desalination system at T = 15 °C and T = 33 °C, corresponding to typical seawater inlet temperatures during winter and summer, respectively. In Figure 4, we see the same overall pattern for exergy destruction in both summer and winter. The model predicts that moving from winter to summer conditions, there would be an increase in pump consumption of 1% (from 49% to 50%), due to the changes in fluid properties at the two temperatures. Specifically, at 33 °C, the viscosity of water is noticeably less than at 15 °C, which means an increase in both flow rate and Reynolds number for the same pressure difference. This will increase turbulence with a consequent increase in energy losses in pumps, pipes, and fittings, as well as increased frictional losses, all increasing entropy generation. In contrast, there was a decrease in exergy destruction in the first RO unit from 23% to 21% when changing from winter to summer, as would be expected from the known temperature dependence of membrane transport properties. The higher the water temperature, the greater its permeability due to its lower viscosity and increased molecular diffusivity, which enables higher permeate flux at the same transmembrane pressure with a lower driving force needed for separation, and less irreversibility associated with pressure losses, concentration polarization, and internal diffusion processes. In addition, we note that at higher temperatures, there will be enhanced mass transfer near the membrane surface, which can reduce concentration polarization effects, further reducing entropy generation. In addition, we note that at higher temperatures, there will be enhanced mass transfer near the membrane surface, which can reduce concentration polarization effects, further reducing entropy generation. When considering the feasibility of operating at thermodynamically more favourable higher temperature conditions, the corresponding risk factors must be considered. Increasing feed temperature will increase the rate at which the membrane ages, the likelihood of scaling, and biological fouling.

The contributions to exergy destruction of the second and third RO units remain stable at about 7–8% in both winter and summer, because these stages operate with lower salinity level feeds and experience smaller osmotic pressure differences. Making their performance less dependent on temperature. Similarly, the ERD shows very little change because its performance is driven primarily by the efficiency of pressure exchange rather than by the thermophysical properties of the working fluids, although small changes due to viscosity-related frictional effects may still occur. The valves show a very slight increase in exergy destruction in the summer, primarily due to increased flow, which results in greater mixing and higher local turbulence intensity and viscous energy dissipation. The contributions of the remaining components, including the strainers, remain nearly constant. Overall, we see that the difference in temperatures between summer and winter has a major effect on hydraulic irreversibilities in the pumping system and mass transfer related irreversibilities in the membrane units. The lower winter temperatures reduced membrane performance but produced slightly lower mechanical losses in the pumping system.

**Figure 5:**
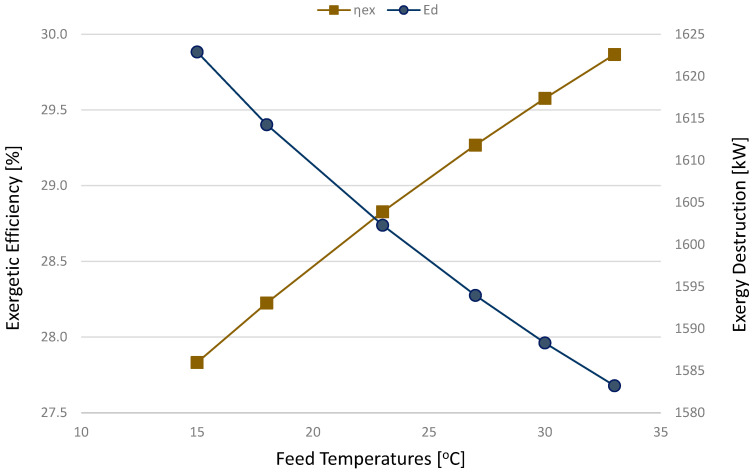
Exergetic efficiency and exergy destruction of the RO desalination unit as a function of seawater feed temperature.

Figure 5 shows how the temperature of the feed water affects the overall exergetic efficiency and total exergy destruction of the triple pass reverse osmosis seawater desalination system for typical winter and summer inlet seawater temperatures (15 °C to 33 °C). The two parameters clearly show opposite trends. A higher feed temperature raises the exergetic efficiency monotonically from 27.8% at 15 °C to 29.9% at 33 °C, showing that the higher the inlet feed temperature, the lower the thermodynamic losses in the system. This is due primarily to the enhanced water permeability of the membrane at elevated temperatures due to increased molecular diffusivity and reduced solution viscosity, enabling the same permeate flux to be obtained at a lower transmembrane pressure. This improvement in membrane permeability translates directly into higher permeate production at elevated feed temperatures. The reduction in irreversibilities associated with lower pressure losses due to the reduced driving force, together with concentration polarization and internal diffusion across the membrane active layer, all result in a more thermodynamically efficient separation process. However, the exergy destruction decreases steadily from 1622 kW to 1582 kW when the inlet seawater temperature rises from 15 °C to 33 °C, confirming that the higher the feed water temperature, the lower the system’s overall thermodynamic irreversibilities. This is achieved primarily by improving membrane performance in the first RO unit, the second largest source of exergy destruction. The reverse correspondence of exergetic efficiency and exergy destruction is fully consistent, thermodynamically, with a lower level of irreversibilities translating directly into a greater proportion of the exergy input being useful separation work. These results offer a thermodynamic explanation for the trend in modern large-scale desalination plants toward hybridization of reverse osmosis systems using thermal desalination technology, such as multi-stage flash and multi-effect distillation. Such hybrid configurations feed the warmed brine or reject stream discharges from the thermal desalination units (typically 30–45 °C) to the RO system, raising the RO feeding temperature above that of ambient seawater. The results show that a higher feed temperature improves the plant’s overall thermodynamic performance by directly increasing the exergetic efficiency and simultaneously lowering the exergy destruction of the RO system. A warmer feed reduces the osmotic pressure resistance of the feed stream, lowering the specific energy consumption of the high-pressure RO pumps, and thus reducing both thermodynamic irreversibilities and operating costs. However, hybrid RO-thermal integration introduces well-known operational tradeoffs, which include increased brine salinity, higher chemical dosing requirements, and additional energy demand on the thermal side. These trade-offs must be carefully evaluated against any thermodynamic gains before implementation. Combining thermodynamic benefits with the benefits of shared intake and outfall infrastructures is a compelling reason to continue improving hybrid RO-thermal desalination systems, particularly for use in regions such as the Red Sea and the Arabian Gulf, where both thermal and membrane technologies are already beneficially employed [24].

**Figure 6:**
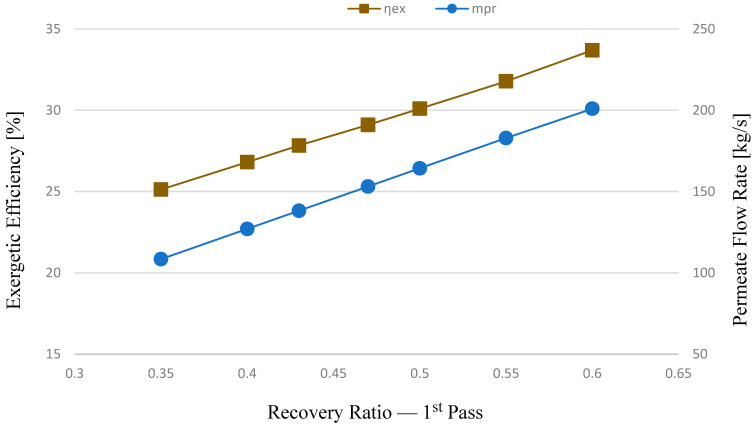
Effect of the first-pass recovery ratio on the exergetic efficiency and permeate flow rate of the reverse osmosis (RO) unit in a triple-pass configuration.

Figure 6 presents the effect of the first-pass recovery ratio (RC_1)_ on the overall exergetic efficiency and permeate flow rate of the triple-pass seawater reverse osmosis desalination system over a recovery ratio range of 0.35 to 0.60. Both exergetic efficiency and permeate flow rate show similar monotonic increases with increase in RC_1_, a sign of the coupled thermodynamic and hydraulic responses of the system to enhanced water extraction in the first pass. The exergetic efficiency increases steadily from 25.1% at RC_1_ = 0.35 to 33.7% at RC_1_ = 0.60, a significant improvement of some 8.6% over the given range, which is attributed to increased utilization of the available exergy input at higher values of the first-pass recovery ratio. The ratio of useful separation work output to total exergy input increases as more permeate is extracted per unit of feed indicating an improvement in the overall system thermodynamic efficiency. Further, the ERD will operate more efficiently at higher recovery ratio, recovering a higher proportion of the hydraulic exergy in the stream of high-pressure concentrate and recirculating it as useful energy to the feed stream. This will further reduce exergy destruction and increase system efficiency. At the same time, permeate flow rate increased linearly from 108 kg/s at RC_1_ = 0.35 to 201 kg/s at RC_1_ = 0.60, an 86% increase in product water output. The near linear relationship between the permeate flow rate and the first-pass recovery ratio confirms that permeate production is approximately directly proportional to the recovery ratio under the given operating conditions. It also means that the second and third passes have the same proportionate response to the increased feed from the first pass. Both exergetic efficiency and permeate flow rate show the same rate of increase with RC_1_, which is a very significant operationally, as it shows that greater recovery will simultaneously improve both thermodynamic performance and water production, without incurring a thermodynamic penalty. Such findings imply that if concentrate scaling risk, equipment pressure limits, and membrane fouling can be suitably managed, then operating a triple pass Reverse Osmosis system at an RC_1_ value of 0.6, the upper end of the recovery ratio range investigated is thermodynamically favorable. The results confirm RC_1_ as the primary control parameter for optimizing both energy efficiency and production capacity of triple pass reverse osmosis seawater desalination systems.

**Figure 7:**
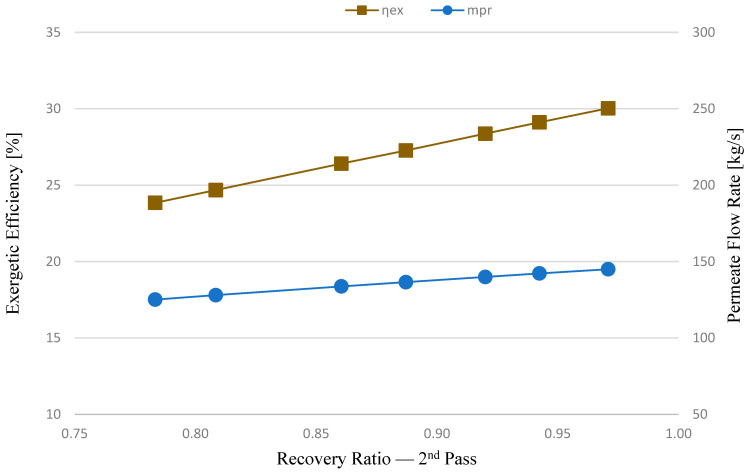
Effect of the second-pass recovery ratio on the exergetic efficiency and permeate flow rate of the reverse osmosis (RO) unit in a triple-pass configuration.

Figure 7 presents the effect of the second-pass recovery ratio (RC_2_) on the overall exergetic efficiency and permeate flow rate for 0.78 ≤ RC_2_ ≤ 0.98. We see both the overall exergetic efficiency and permeate flow rate follow a linearly increasing trend, see also Figure 5, again demonstrating that higher recovery improves thermodynamic performance and product water output, regardless of which pass is considered. The exergetic efficiency increases from 23.9% at RC_2_ = 0.78 to 30.0% at RC_2_ = 0.98, an improvement of 6.1%, compared with 8.6% achieved by changing RC_1_. However, the permeate flow rate increases from about 125 kg/s to 145 kg/s, which is a gain of just 16% compared to the 86% increase seen in Figure 5. The inherently lower osmotic pressure and reduced salinity of the second pass feed stream limit the thermodynamic impact of variations in RC_2_. Thus, the small improvements seen in Figure 7 are consistent with the first pass (RC_1_) exerting a much greater influence on both system exergetic efficiency and total permeate production, confirming RC_1_ as the dominating parameter when optimizing the thermodynamic performance of a triple pass RO.

**Figure 8:**
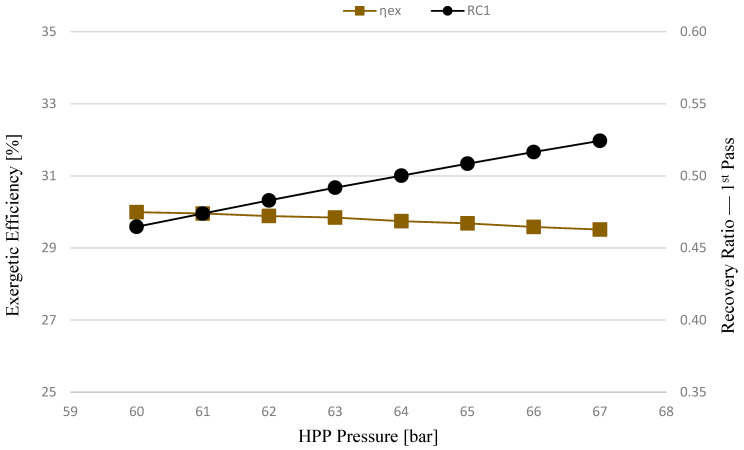
Effect of high-pressure pump operating pressure on exergetic efficiency and recovery ratio of the first pass in a triple-pass reverse osmosis desalination system.

Figure 8 presents the effect of the high-pressure pump (HPP) operating pressure on the overall exergetic efficiency and first-pass recovery ratio of the triple-pass reverse osmosis seawater desalination system over a pressure range of 60 to 67 bar. The two parameters show opposing trends with increasing HPP pressure, revealing a trade-off between water production and energy utilization efficiency. With an increase in pump operating pressure from 60 bar to 67 bar in steps of 1 bar, the first pass RC_1_ increased monotonically from 0.46 to 0.52, as expected, given that the relationship between applied hydraulic pressure and net driving pressure across the membrane is well established. As the HPP pressure increases, so does the gap between the net driving pressure and the osmotic pressure resisting the feed stream. Thus, there is an increase in the water forced through the membrane, increasing permeate extraction. However, unlike the previous experiments, the results of which are shown in Figure 6 and Figure 7, where HPP pressure was held constant and recovery ratio was varied independently, in the present case, we have both RC_1_ and exergetic efficiency responding simultaneously to changes in the pressure delivered by the HPP. The distinction is thermodynamically significant. In Figure 5 and Figure 6, with the operating pressure fixed, the gains in exergetic efficiency obtained by increasing RC_1_ and RC_2_ were achieved without incurring further pressure-related irreversibilities. However, when RC_1_ increases as a result of increasing the HPP pressure, the extra exergy input needed to raise the pressure grows at a faster rate than the gain in useful separation work. Consequently, the exergetic efficiency declines slightly from 29.99% at 60 bar to 29.51% at 67 bar, because rather than being converted into productive separation work, the extra exergy input is dissipated as irreversibilities within the membrane modules, pressure vessels, and associated flow components.

Thus, HPP pressure and recovery ratio will have distinctly different thermodynamic consequences when varied independently than when varied simultaneously. It follows that operating at the minimum HPP pressure to attain the target recovery ratio is the thermodynamically optimal strategy for a triple-pass RO system, avoiding the unnecessary exergy destruction associated with overpressurization.

**Figure 9:**
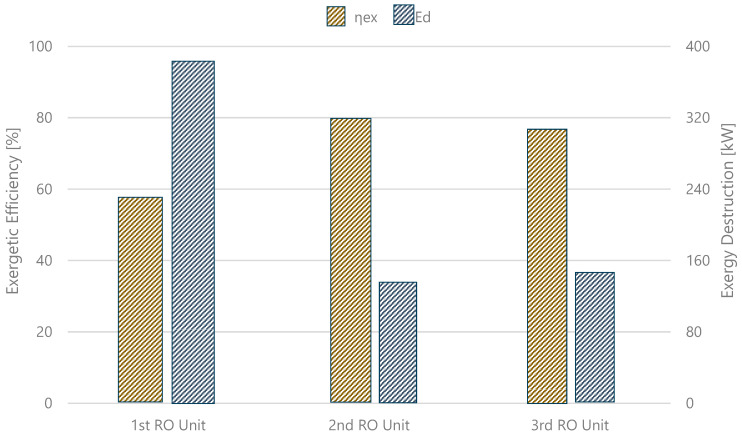
Exergetic efficiency and exergy destruction of each reverse osmosis unit in a triple-pass seawater desalination system.

Figure 9 shows the overall exergetic efficiency and exergy destruction for each of the three RO units in a triple pass reverse osmosis seawater desalination system. The results show clearly that with an increase in the exergetic efficiency across the three passes, there is a corresponding decrease in exergy destruction. This provides thermodynamic insight into system performance at the unit level. The first RO unit shows the lowest exergetic efficiency (about 57%) with the highest exergy destruction (about 375 kW). This is what would be expected since the first pass processes the seawater feed at its highest salinity and contamination level, and operating pressure, with the result that there will be the greatest osmotic pressure resistance, concentration polarization, and hydraulic irreversibilities. The second RO unit, operating at a feeding pressure of 8.8 bar, shows a very much increased exergetic efficiency level (about 80%) and very much reduced exergy destruction (about 120 kW). This marked improvement relative to the first pass is attributed to the considerably lower feed salinity and pollution levels entering the second pass. This substantially reduces both the necessary transmembrane driving force and osmotic pressure resistance, reducing pressure-related irreversibilities and concentration polarization effects. The third RO unit, operating at 9.0 bar, shows an exergetic efficiency of 78% and an exergy destruction of 130 kW. This unit is processing a feed of even lower salinity than the second unit, but at a slightly higher operating pressure, resulting in slightly more hydraulic irreversibilities and explaining the minor reduction in exergetic efficiency from 80% to 78%. We see from the data for passes two and three that, under low salinity conditions, small increases in operating pressure can noticeably affect thermodynamic performance. The increase in the exergetic efficiency from first to second/third RO units reflects the advantage of the multi-pass configuration, wherein successive passes operate under progressively milder separation conditions.

**Figure 10:**
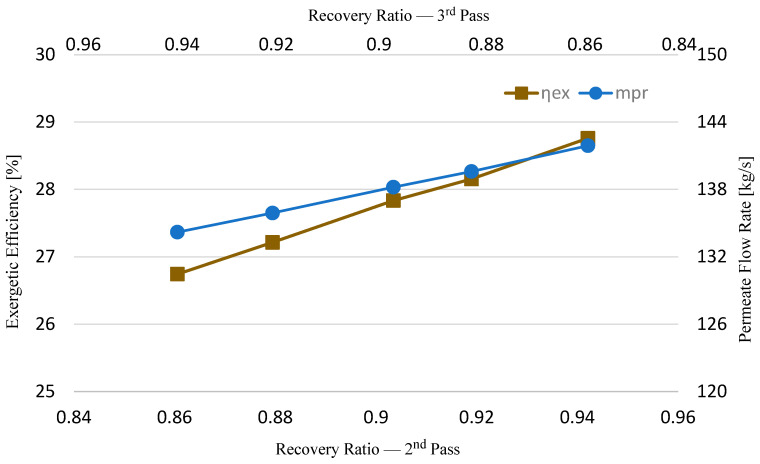
Effect of varying recovery ratio of the 2nd and 3rd passes on system exergetic efficiency and permeate flow rate.

Figure 10 shows the effects of the recovery ratios for the second and third passes on the overall exergetic efficiency and permeate flow rate of the triple-pass reverse osmosis seawater desalination plant. The lower *x*-axis represents the value of RC_2_, in the range 0.86 ≤ RC_2_ ≤ to 0.94, while the upper *x*-axis represents value of RC_3_, in the range 0.94 ≥ RC_2_ ≥ 0.86, reflecting the constraint that the total system recovery is held constant while RC_2_ and RC_3_ are varied simultaneously in opposing directions. Both η_ex_ and *ṁ_pr_* increase consistently with an increase in RC_2_: with RC_2_ = 0.86 and *ṁ_pr_* = 134 kg/s, the value of η_ex_ is 26.7%, and when RC_2_ = 0.94 and *ṁ_pr_* = 142 kg/s, the value of η_ex_ is 28.9%, improvements of 2.2% and 6.0%, respectively. These trends are consistent with the results presented in Figure 6 and Figure 7, which show that higher values of recovery ratios improve *η_ex_* and *ṁ_pr_* by increasing the ratio of useful separation work to total exergy input. However, the magnitude of improvement observed here is considerably less than that observed for RC_1_ in Figure 6, which is what would be expected given the already low values of salinity and operating pressure of the second and third passes. The changes in RC_3_ with RC_2_ are in opposite directions. When RC_2_ increases, RC_3_ decreases and vice versa, which means that the permeate extracted by the third pass gets smaller as that extracted by the second gets larger, as though the two were competing thermodynamically. However, the overall η_ex_ and ṁ_pr_ continue to increase, showing that the thermodynamic gain from an improved second pass recovery will be greater than the thermodynamic penalty incurred in the third pass recovery. It follows that for the second and third passes, RC_2_ is the more important for optimization at the system level; this is consistent with its higher operating pressure of 8.8 bar compared to that of the third pass at 9.0 bar, see Figure 9. The results presented in Figure 6, Figure 7 and Figure 10 provide an order of importance across the three passes, with RC_1_ having the greatest thermodynamic effect, followed by RC_2_, with RC_3_ having the least effect.

**Figure 11:**
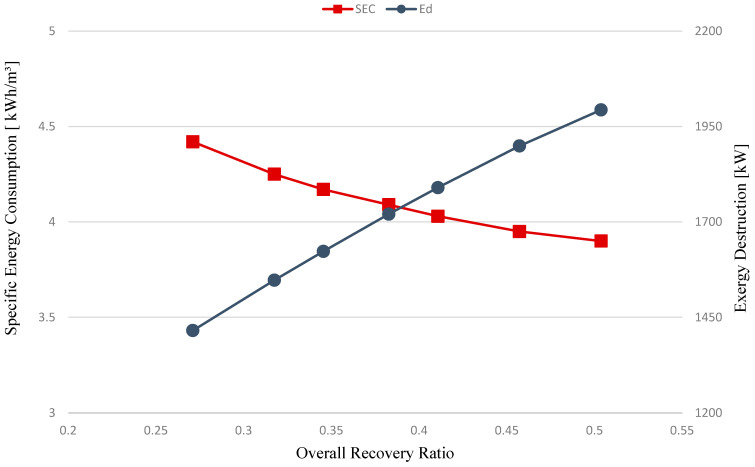
Effect of overall recovery ratio on specific energy consumption and exergy destruction of a triple-pass SWRO desalination system.

Figure 11 presents the specific energy consumption and total exergy destruction of the TPRO seawater desalination system as a function of the overall recovery ratio in the range 0.27 to 0.51. The two parameters clearly show opposing trends, indicating a fundamental trade-off between specific energy consumption (SEC), a measure of energy efficiency, and exergy destruction (irreversibilities) as system recovery increases. Figure 11 shows that the SEC decreases steadily from 4.42 kWh/m^3^ at an RC value of 0.27 to 3.90 kWh/m^3^ at an RC value of 0.51. As discussed above, this is due primarily to the ERD, which recovers proportionally more hydraulic energy from the stream of high-pressure concentrate at higher RC values, but also because the fixed auxiliary energy load is spread over a larger permeate output. This response is markedly different from that of conventional single-pass SWRO systems, where, typically, the SEC increases with RC beyond the optimal point. This further emphasizes the thermodynamic advantage of a triple-pass configuration equipped with an ERD.

We see exergy destruction (Ėd) increases steadily from 1416 kW at an RC = 0.27 to 1994 kW at an RC = 0.51. This reflects the increase in thermodynamic irreversibilities (which generate greater entropy) within the system at higher values of RC, when the first-pass HPP must operate at the higher pressures required to overcome the osmotic resistance associated with the more concentrated feed stream. The simultaneous increase in Ėd and decrease in SEC, with an increase in RC values, confirms that, while the system may be more energy-efficient in terms of SEC, the absolute magnitude of thermodynamic losses increases due to the higher exergy input required to sustain elevated recovery operation.

The SEC and Ėd trends intersect at an RC value of 0.39, corresponding to SEC and Ėd values of 4.05 kWh/m^3^ and 1700 kW respectively. While this does not represent the optimum operating point, it does identify a practical compromise between minimizing SEC and limiting exergy destruction. As RC increases beyond 0.39, any further reduction in SEC is accompanied by progressively higher exergy destruction, highlighting the trade-off between energy efficiency and thermodynamic irreversibility. Therefore, RC = 0.39 may be considered a practical operating target for the investigated triple-pass RO system. We note that the preferred operating point will be plant-specific depending on the given objectives and economic considerations. A formal thermo-economic optimization incorporating site-specific cost parameters is recommended for future work.

## 6. Conclusions

A comprehensive energy and exergy analysis of a triple-pass reverse osmosis seawater desalination system has been carried out using IPSEpro which showed good agreement when validated against manufacturer-provided data. The exergetic efficiency of the RO units increases between the first and successive passes, from 57% in the first pass to 80% in the second and 78% in the third. The enhancement is due to the reduced osmotic pressure and lower operating pressures in the later stages. Considering the different components, we see that the pumping system is the source of the largest exergy destruction (49%), primarily due to the main HPP, followed by the first RO unit (23%). It follows that these are the key components for performance improvement. Feed water temperature, as expected, has a noticeable impact on system performance, mainly due to improved membrane permeability. These results support considering the integration of RO systems with thermal desalination technologies in hybrid configurations, where higher feed temperatures can improve thermodynamic performance. Of the operating parameters, the recovery ratio of the first pass has the greatest influence on system performance. In contrast, variations in the recovery ratio of the 2nd and 3rd passes have a comparatively little effect. This confirms the desirability of operating at the minimum pressure required to achieve the desired recovery ratio. An operating point has been identified with an overall recovery ratio of 0.39, representing the optimal balance between energy efficiency and thermodynamic performance. Overall, system performance can be improved by reducing exergy destruction through the use of high-efficiency variable-speed pumps with variable frequency drives, enhanced membrane pretreatment and cleaning strategies, and advanced energy recovery devices with lower internal losses. Future work will extend this framework to incorporate brine exergy losses, economic assessment, and environmental impact analysis.

## Figures and Tables

**Table 1:** Exergy destruction rates and exergetic efficiencies of the key components in the proposed system under steady state conditions.

No.	Component	Schematic	Exergy Destruction ${\dot{E}}_{d}$	Exergetic Efficiency $\eta_{ex}$
1	Membrane	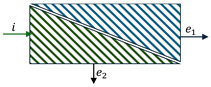	$\left({\dot{E}}_{i}-{\dot{E}}_{e2}\right)-{\dot{E}}_{e1}$	$\frac{{\dot{E}}_{e1}}{{\dot{E}}_{i}-{\dot{E}}_{e2}}$
2	Pump	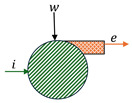	${\dot{E}}_{w\;}-\left({\dot{E}}_{e}-{\dot{E}}_{i}\right)$	$\frac{{\dot{E}}_{e}-{\dot{E}}_{i}}{{\dot{E}}_{w\;}}$
3	Mixing Unit	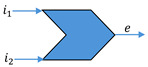	$\left({\dot{E}}_{i1}+{\dot{E}}_{i2}\right)-{\dot{E}}_{e}$	$\frac{{\dot{E}}_{e}}{{\dot{E}}_{i1}+{\dot{E}}_{i2}}$
4	Splitter Unit	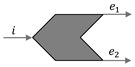	${\dot{E}}_{i}-\left({\dot{E}}_{e1}+{\dot{E}}_{e2}\right)$	$\frac{{\dot{E}}_{e1}+{\dot{E}}_{e2}}{{\dot{E}}_{i}}$
5	Pressure Exchanger	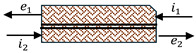	$\left({\dot{E}}_{i1}+{\dot{E}}_{i2}\right)-\left({\dot{E}}_{e1}+{\dot{E}}_{e2}\right)$	$\frac{{\dot{E}}_{e1}+{\dot{E}}_{e2}}{{\dot{E}}_{i1}+{\dot{E}}_{i2}}$
6	Expansion Valve	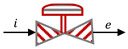	${\dot{E}}_{i}-{\dot{E}}_{e}$	$\frac{{\dot{E}}_{e}}{{\dot{E}}_{i}}$

**Table 2:** Model validation against manufacturer performance test data for the triple-pass RO desalination plant.

Description		Manufacturer Data ^(a)^	Proposed Model ^(b)^	Unit	Deviation [%]
First Pass	Feed flow rate	1327	1305	(m^3^/h)	1.66
	Permeate flow rate	570	561.6	(m^3^/h)	1.47
	Brine/reject flow rate	767	743	(m^3^/h)	3.13
	Feed conductivity	59,000	59,000	(µS/cm)	----
	Permeate conductivity	280	285.8	(µS/cm)	2.07
	Operating pressure	64	64	(kgf/cm^2^)	----
	Recovery ratio	43	43	(%)	0.00
Second Pass	Feed flow rate	626	617.5	(m^3^/h)	1.36
	Permeate flow rate	564	557.9	(m^3^/h)	1.08
	Brine/reject flow rate	62	59.6	(m^3^/h)	3.87
	Feed conductivity	280	285.8	(µS/cm)	2.07
	Permeate conductivity	26	25.9	(µS/cm)	0.38
	Operating pressure	9	9	(kgf/cm^2^)	----
	Recovery ratio	90	90.4	(%)	0.44
Third Pass	Feed flow rate	564	557.9	(m^3^/h)	1.08
	Permeate flow rate	508	502.1	(m^3^/h)	1.16
	Brine/reject flow rate	56	55.8	(m^3^/h)	0.36
	Feed conductivity	26	25.9	(µS/cm)	0.38
	Permeate conductivity	11	11.3	(µS/cm)	2.73
	Operating pressure	10	10	(kgf/cm^2^)	----
	Recovery ratio	90	90	(%)	0.00
Seawater inlet flow		1265	1297	(m^3^/h)	2.53
Permeate flow rate		508	502.1	(m^3^/h)	1.16
Brine discharge		767	763.1	(m^3^/h)	0.51

^a^ MHI [35], ^b^ Present work calculations.

**Table 3:** Thermodynamic properties at various points within the RO system.

No.	$\dot{m}$ kg/s	T °C	P Bar	$w_{s}$ PPM	e kJ/kg	E_x_ kW
0	400.0	15.00	1.0	41,000	0.000	0.0
1	400.0	15.01	2.0	41,000	0.097	38.7
2	370.0	15.99	1.6	41,000	0.052	19.2
3	365.0	15.00	1.9	41,000	0.089	32.6
4	355.0	14.98	1.5	41,000	0.053	18.7
5	10.0	14.99	1.1	41,000	0.008	0.1
6	164.8	14.65	1.4	39,204	0.043	7.0
6A	207.2	14.65	1.4	39,204	0.043	8.8
7	164.8	15.19	63.0	39,204	6.021	992.2
7A	207.8	14.75	63.0	39,501	6.025	1252.0
8	372.6	14.94	63.0	39,370	6.023	2244.1
9	160.2	14.94	1.1	186	3.075	492.6
10	212.4	14.94	62.0	68,929	6.486	1377.5
10A	207.8	14.72	59.1	39,501	5.651	1174.2
11	211.8	14.99	1.2	68,719	0.701	148.4
12	176.1	15.33	1.0	178	3.048	536.7
13	176.1	15.41	8.8	178	3.847	677.4
14	17.0	15.41	7.8	1695	3.466	58.9
15	159.1	15.41	1.0	16	3.098	492.9
16	159.1	15.49	9.8	16	3.978	632.9
17	143.2	15.49	1.2	7	3.120	446.8
18	15.9	15.49	4.0	98	3.383	53.8
19	143.2	15.00	1.1	7	3.107	444.9
20	143.2	15.01	2.5	7	3.249	465.2
21	138.2	15.01	2.5	7	3.249	449.0
23	5.0	15.01	2.5	7	3.249	16.2
24	10.0	15.01	2.0	41,000	0.097	1.0
25	10.0	15.99	1.6	41,000	0.052	0.5
26	10.0	16.00	2.0	41,000	0.091	0.9
27	217.8	14.99	1.1	68,000	0.662	144.2
28	10.0	15.00	2.5	41,000	0.146	1.5
29	6.0	15.00	2.0	41,000	0.097	0.6
30	4.0	15.00	2.0	41,000	0.097	0.4

## Data Availability

The original contributions presented in the study are included in thearticle, further inquiries can be directed to the corresponding author.

## Abbreviations

**Nomenclature**			
$\dot{E}$	Rate of exergy flow in the stream	s	Salt
${\bar{e}}_{k}^{ch}$	Specific molar chemical exergy	sw	Seawater
g	Specific Gibbs energy	w	pure water
h	Specific enthalpy	x	Total
$h_{fg}$	Latent heat of vaporization	**Abbreviations**	
M	Molar mass	BWRS	Brackish water reverse osmosis
$\dot{m}$	Mass flow rate	DAF	Dissolved air flotation
$\dot{n}$	Number of moles	EBP	ERD booster pump
P	Pressure	FFP	Filter feed pump
$\dot{Q}$	Heat transfer rate	FWRS	Freshwater reverse osmosis
*RC*	Recovery ratio	ERD	Energy recovery device
s	Specific entropy	HPP	High-pressure pump
$\dot{S}$	Entropy generation	MED	Multi-effect desalination
t	Time	MSF	Multi-stage flash
T	Temperature	PWP	Product water pump
y	Mole fraction	RO	Reverse osmosis
v	Specific volume	SEC	Specific energy consumption
$W_{min}$	Minimum work of separation	SWRO	seawater reverse osmosis
$w_{s}$	Salinity	TDS	Total dissolved solids
**Greek letters**		TPRO	Triple-Pass Reverse Osmosis
$\eta_{ex}$	Exergetic efficiency	UF	Ultrafiltration
Δ	Difference	UFP	Ultrafiltration pump
ρ	Density	WCP	Water cooling pump
μ	Chemical potential	WWP	Waste water pump
**Subscripts**			
1, 2	Substances 1 and 2		
ac	Actual		
c	Cold stream		
ch	Chemical		
cv	Control volume		
d	Destruction		
e	Outlet		
f	Fuel		
fr	Feed		
h	Hot Stream		
i	Inlet		
k	Component		
ke	Kinetic energy		
L	Loss		
o	Environmental dead state		
p	Product		
pr	permeate		
ph	Physical		
pe	Potentials		

## Appendix A. Seawater Thermodynamics Properties

This appendix presents the thermophysical property correlations of Nayar et al. [34] used in the present study, expressed as functions of temperature T (°C) and salinity w_s_.

### Appendix A.1. Density

The density of seawater $\rho_{sw}$ (kg/m^3^) is expressed as a function of temperature T (°C) and salinity $w_{s}$ (kgs/kgsw). The correlation proposed by [34] accounts for the contribution of dissolved salts to the bulk density through a set of empirically derived coefficients, and is given by:

(A1) $$\rho_{sw}\left(t,w_{s},P\right)={\rho_{sw}\left(t,w_{s},P_{o}\right)\times F}_{p}$$

where,

(A2) $$\rho_{sw}\left(t,ws,P_{o}\right)=\left(a_{1}+a_{2}T+a_{3}T^{2}+a_{4}T^{3}+a_{5}T^{4}\right)+(b_{1}{ws}_{kgs/kgsw}+b_{2}{ws}_{kgs/kgsw}T\;{+b_{3}{{ws}_{kgs/kgsw}T}^{2}+b}_{4}{{ws}_{kgs/kgsw}T}^{3}+b_{5}{{w^{2}s}_{kgs/kgsw}T}^{2})$$

(A3) $$F_{p}=\exp\left[\begin{array}{c} \left(P-P_{o}\right)\times \left(c_{1}+c_{2}T+c_{3}T^{2}+c_{4}T^{3}+c_{5}T^{4}+c_{6}T^{5}+{ws}_{gs/kgsw}\times \left(a_{1}+a_{2}T+a_{3}T^{2}\right)\right)\\ +\frac{\left(P^{2}-P_{o}^{2}\right)}{2}\times \left(c_{7}+c_{8}T+c_{9}T^{3}+d_{4}{ws}_{gs/kgsw}\right) \end{array}\right]$$

The empirical constants appearing in Equations (A2) and (A3), which are required for the evaluation of seawater density over the applicable ranges of temperature and salinity, are summarized in Table A1.

### Appendix A.2. Specific Enthalpy

The specific enthalpy of seawater $h_{sw}$ (kJ/kg) is expressed as a function of temperature T (°C) and salinity $w_{s}$ (gs/kgsw) or (kgs/kgsw). Following the correlation proposed by [34], the specific enthalpy accounts for the thermal energy contribution of both pure water and dissolved salts, and is defined as:

(A4) $$h_{sw}\left(t,w_{s},P\right)=h_{sw}\left(t,ws,P_{o}\right)+\left(P-P_{o}\right)\times \left(a_{1}+a_{2}T+a_{3}T^{2}+a_{4}T^{3}+{w_{s}}_{gs/kgsw}\times (a_{5}+a_{6}T+a_{7}T^{2}+a_{8}T^{3})\right)$$

where,

(A5) $$h_{sw}\left(t,ws,P_{o}\right)=h_{w}\left(t\right)-{w_{s}}_{kgs/kgsw}\left(b_{1}+b_{2}{w_{s}}_{kgs/kgsw}+b_{3}{w_{s}^{2}}_{kgs/kgsw}+b_{4}{w_{s}^{3}}_{kgs/kgsw}+b_{5}T+b_{6}T^{2}+b_{7}T^{3}+b_{8}{w_{s}}_{kgs/kgsw}T+b_{9}{w_{s}^{2}}_{kgs/kgsw}T+b_{10}{w_{s}}_{kgs/kgsw}T^{2}\right)$$

(A6) $$h_{w}\left(t\right)=141.355+4202.070T-0.535T^{2}+0.004T^{3}$$

The empirical constants appearing in Equations (A4) and (A5), which are required for the evaluation of the specific enthalpy of seawater over the applicable ranges of temperature and salinity, are summarized in Table A1.

### Appendix A.3. Specific Entropy

The specific entropy of seawater $s_{sw}$ (kJ/kg·K) is expressed as a function of temperature T (°C) and salinity $w_{s}$ (gs/kgsw) or (kgs/kgsw). In accordance with the correlation proposed by [33], the specific entropy captures the degree of thermodynamic disorder arising from both the pure water component and the dissolved salt content, and is defined as:

(A7) $$s_{sw}\left(t,w_{s},P\right)=s_{sw}\left(t,ws,P_{o}\right)+\left(P-P_{o}\right)\times \left(a_{1}+a_{2}T+a_{3}T^{2}+a_{4}T^{3}+{w_{s}}_{gs/kgsw}\times (a_{5}+a_{6}T+a_{7}T^{2}+a_{8}T^{3})\right)$$

where,

(A8) $$s_{sw}\left(t,w_{s},P_{o}\right)=s_{w}\left(t,P_{o}\right)-{w_{s}}_{kgs/kgsw}\left(b_{1}+b_{2}{w_{s}}_{kgs/kgsw}+b_{3}{w_{s}^{2}}_{kgs/kgsw}+b_{4}{w_{s}^{3}}_{kgs/kgsw}+b_{5}T+b_{6}T^{2}+b_{7}T^{3}+b_{8}{w_{s}}_{kgs/kgsw}T+b_{9}{w_{s}^{2}}_{kgs/kgsw}T+b_{10}{w_{s}}_{kgs/kgsw}T^{2}\right)$$

(A9) $$s_{w}=0.1543+15.383T-2.996x10^{-2}T^{2}+8.193x10^{-5}T^{3}-1.37x10^{-7}T^{4}$$

The empirical constants appearing in Equations (A7) and (A8), which are required for the evaluation of the specific entropy of seawater over the applicable ranges of temperature and salinity, are summarised in Table A1.

### Appendix A.4. Gibbs Free Energy

The specific Gibbs free energy of seawater $g_{sw}$ (kJ/kg) is expressed as a function of temperature T (°C) and salinity $w_{s}$ (gs/kgsw) or (kgs/kgsw). The specific Gibbs free energy is a fundamental thermodynamic potential that governs the direction of spontaneous processes and plays a critical role in quantifying the minimum work of separation in desalination systems. Following the correlation proposed by [34], it is defined as:

(A10) $$g_{sw}\left(t,w_{s},P\right)=g_{sw}\left(t,ws,P_{o}\right)+\left(P-P_{o}\right)\times \left(a_{1}+a_{2}T+a_{3}T^{2}+a_{4}T^{3}+{w_{s}}_{gs/kgsw}\times (a_{5}+a_{6}T+a_{7}T^{2}+a_{8}T^{3})\right)$$

where,

(A11) $$g_{sw}\left(t,w_{s},P_{o}\right)=g_{w}\left(t,P_{o}\right)+{w_{s}}_{kgs/kgsw}\left(b_{1}+b_{2}T+b_{3}T^{2}+b_{4}{w_{s}^{2}}_{kgs/kgsw}T+b_{5}{w_{s}^{2}}_{kgs/kgsw}T^{2}+b_{6}{w_{s}^{3}}_{kgs/kgsw}+b_{7}{w_{s}^{3}}_{kgs/kgsw}T^{2}+b_{8}{{Ln(w}_{s}}_{kgs/kgsw})+b_{9}{{Ln(w}_{s}}_{kgs/kgsw})T\right)$$

(A12) $$g_{w}\left(t,P_{o}\right)=c_{1}+c_{2}T+c_{3}T^{2}+c_{4}T^{3}+c_{5}T^{4}$$

The empirical constants appearing in Equations (A10)–(A12), which are required for the complete evaluation of the specific Gibbs free energy of seawater over the applicable ranges of temperature and salinity, are summarized in Table A1.

**Table A1:** Constants used in empirical correlations [34].

Constants of density Equations (A2) and (A3)	a_1_ = 8.02 × 10^2^	b_1_ = −2.348 × 10^4^	c_1_ = −4.231 × 10^2^	c_6_ = −4.231 × 10^2^	d_2_ = −1.443 × 10^−1^
	a_2_ = −2.001 × 10^1^	b_2_ = 3.152 × 10^5^	c_2_ = 1.463 × 10^4^	c_7_ = 1.463 × 10^4^	d_3_ = 5.879 × 10^−4^
	a_3_ = 1.677 × 10^−2^	b_3_ = 2.803 × 10^6^	c_3_ = −9.88 × 10^4^	c_8_ = −9.88 × 10^4^	d_4_ = −6.111 × 10^1^
	a_4_ = −3.06 × 10^−5^	b_4_ = −1.446 × 10^7^	c_4_ = 3.095 × 10^5^	c_9_ = 3.095 × 10^5^	**-----**
	a_5_ = −1.613 × 10^−5^	b_5_ = 7.826 × 10^3^	c_5_ = 2.562 × 10^1^	d_1_ = 2.562 × 10^1^	**-----**
Constants of specific enthalpy Equations (A4) and (A5)	a_1_ = 996.7767	a_5_ = −1.1745	b_1_ = −2.34825 × 10^4^	b_5_ = 7.82607 × 10^3^	b_9_ = 2.77846 × 10^4^
	a_2_ = −3.2406	a_6_ = 0.01169	b_2_ = 3.15183 × 10^5^	b_6_ = −4.41733 × 10^1^	b_10_ = 9.72801 × 10^1^
	a_3_ = 0.01270	a_7_ = −2.6185 × 10^−5^	b_3_ = 2.80269 × 10^6^	b_7_ = 2.13940 × 10^−1^	**-----**
	a_4_ = −4.7723 × 10^−5^	a_8_ = 7.0661 × 10^−8^	b_4_ = −1.44606 × 10^7^	b_8_ = −1.99108 × 10^4^	**-----**
Constants of specific entropy Equations (A7) and (A8)	a_1_ = −4.4786 × 10^−3^	a_5_ = −1.5531 × 10^−3^	b_1_ = −4.231 × 10^2^	b_5_ = 2.562 × 10^1^	b_9_ = 8.041 × 10^1^
	a_2_ = −1.1654 × 10^−2^	a_6_ = 4.0054 × 10^−5^	b_2_ = 1.463 × 10^4^	b_6_ = −1.443 × 10^−1^	b_10_ = 3.035 × 10^−1^
	a_3_ = 6.1154 × 10^−5^	a_7_ = −1.4193 × 10^−7^	b_3_ = −9.880 × 10^4^	b_7_ = 5.879 × 10^−4^	**-----**
	a_4_ = −2.0696 × 10^−7^	a_8_ = 3.3142 × 10^−10^	b_4_ = 3.095 × 10^5^	b_8_ = −6.111 × 10^1^	**-----**
Constants of specific enthalpy Equations (A10), (A11) and (A12)	a_1_ = 996.1978	a_6_ = 1.5712 × 10^−3^	b_3_ = 7.4761 × 10^−3^	b_8_ = 6.6448 × 10^1^	c_4_ = 8.3627 × 10^−3^
	a_2_ = 3.4910 × 10^−2^	a_7_ = −1.8919 × 10^−5^	b_4_ = 1.3836 × 10^−3^	b_9_ = 2.0681 × 10^−1^	c_5_ = −7.8754 × 10^−6^
	a_3_ = 4.7231 × 10^−3^	a_8_ = 2.5939 × 10^−8^	b_5_ = −6.7157 × 10^−6^	c_1_ = 1.0677 × 10^2^	**-----**
	a_4_ = −6.9037 × 10^−6^	b_1_ = −2.4176 × 10^2^	b_6_ = 5.1993 × 10^−4^	c_2_ = −1.4303	**-----**
	a_5_ = −7.2431 × 10^−1^	b_2_ = −6.2462 × 10^−1^	b_7_ = 9.9176 × 10^−9^	c_3_ = −7.6139	**-----**
